# Gastrointestinal stromal tumor of the ampulla of Vater: A case report and literature review

**DOI:** 10.1016/j.ijscr.2022.107598

**Published:** 2022-09-07

**Authors:** Wissal Skhiri, Jamal Saad, Ines Mazhoud, Mohamed Maatouk, Asma Chaouch, Amina Ben Salem

**Affiliations:** a Department of Radiology, Fattouma Bourguiba Monastir, Tunisia; b Department of Surgery, Fattouma Bourguiba Monastir, Tunisia

**Keywords:** GISTs, Gastrointestinal stromal tumors, AV, ampulla of vater, STAV, stromal tumor of ampulla of Vater, Gastrointestinal stromal tumor, Ampulla of Vater, Radiology

## Abstract

**Introduction:**

Gastrointestinal stromal tumors (GISTs) usually develop in the stomach and small intestine and only rarely occur in the ampulla of Vater (AV).

**Case presentation:**

We report a case of a GIST of the AV. A 21-year-old, previously healthy woman presented with a three-month history of epigastric pain, jaundice and weight loss. The diagnosis of a tumor in the second part of the duodenum was made by the computed tomography. The patient underwent pancreatoduodenectomy, the operative specimen revealed an intermediate risk group of GIST and thus, the patient didn't take adjuvant therapy.

**Discussion:**

GIST rarely develops in the duodenal ampulla region and it has no specific symptomatology. Radiological investigations play an important role in the diagnosis of Stromal Tumor of the Ampulla of Vater (STAV) mainly computed tomography because STAV has a large size in the majority of cases contrary to adenocarcinoma.

**Conclusion:**

The aim of this study is to report a new case of STAV combined with a systematic review of reported cases published in peer-reviewed journals.

## Highlights

• Duodenal GIST is even rarer representing only 4% of these tumors and in the ampullar of Vater is an extremely scarce entity
• Radiological investigations play an important role in the diagnosis of STAV mainly CT
• The gold standard for treatment of STAV is surgical resection without rupture of the capsule.

## 1 Introduction

The AV can exhibit a variety of neoplasms, such as carcinoma, adenoma, neuroendocrine tumor, gangliocytic paraganglioma and GIST. GISTs are the most common nonepithelial tumors of the gastrointestinal tract and they arise from organs in which Interstitial cell of Cajal are present. The majority of GISTs are located in the stomach (60 %–70 %) and the small intestine (20 %–25 %), with only 4 % occurring in the duodenum [1]. The localization of GIST in the AV is very rare [1]. The aim of this study is to report a new case of stromal tumor of ampulla of Vater (STAV) combined with a systematic review of reported cases published in peer-reviewed journals.

## 2 Case report

**Fig. 1:**
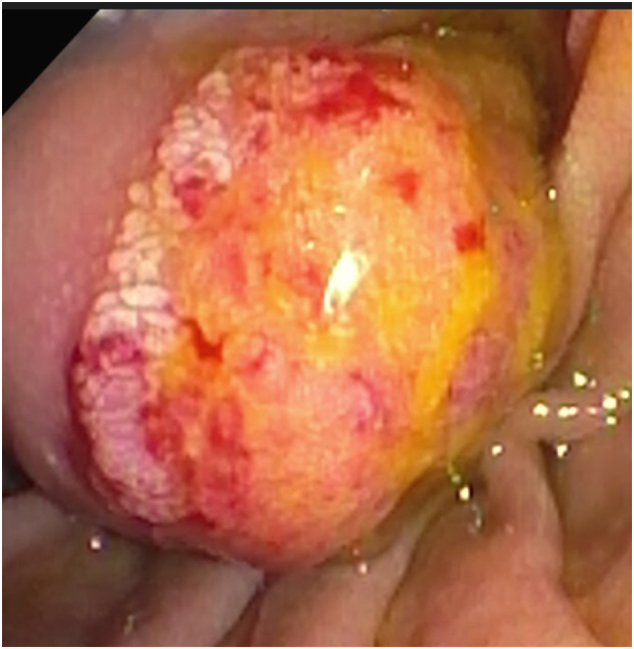
Esophagogastroduodenoscopy showed a bulging ampulla of Vater that bled on touch.

**Fig. 2:**
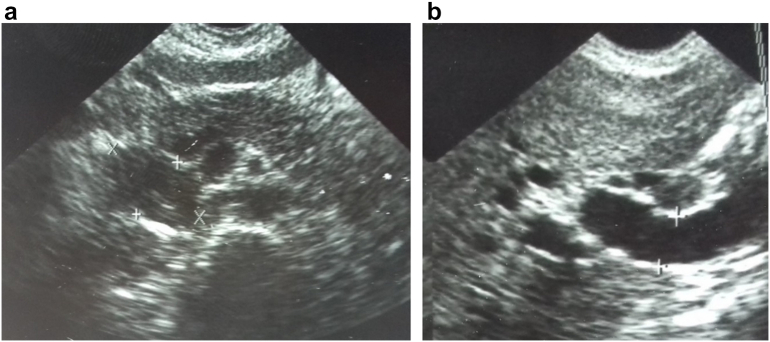
(A, B): Ultrasonography revealed a round, low-echoic mass, responsible for dilated biliary and pancreatic ducts.

**Fig. 3:**
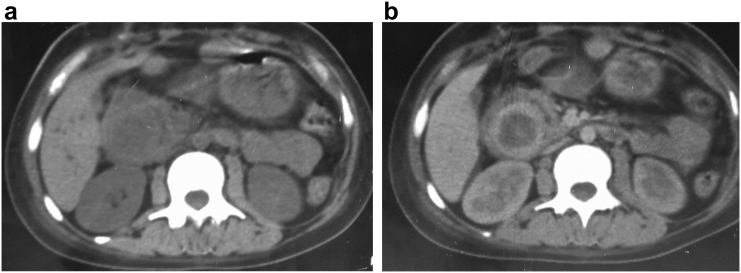
Computed tomography: axial CT scans of the abdomen before (a) and after contrast injection (b) shows a solid and hypodense mass with peripheric enhancement and central necrosis.

A 21 year-old, previously healthy woman, presented with a three-month history of epigastric pain, jaundice and weight loss, accompanied by intermittent black, tarry stools A physical examination revealed pale palpebral conjunctivae and yellowish skin. Her blood pressure was 120/60 mmHg, heart rate was 90 beats/min, and temperature was 36.5 °C. The peripheral blood cell count indicated anemia with a hematocrit of 24 %. Total bilirubin, 7 mg/dL (0.2–1.0 mg/dL); direct bilirubin, 5 mg/dL (0.1–0.5 mg/dL); serum asparate aminotransferase, 100 IU/L (13–30 IU/L); alanine aminotransferase, 200 IU/L (10–40 IU/L); serum lactate dehydrogenase, 430 IU/L (110–210 IU/L); serum alkaline phosphatase, 760 IU/L (100–320 IU/L); serum-glutamyl transpeptidase, 430 IU/L (16–73 IU/L). Esophagogastroduodenoscopy showed a bulging ampulla of Vater that bled on touch. The biopsies were negatives (Fig.1). Ultra-sonography (US) showed a 5 cm hypoechogenic image that had lobulated limits. It was pushing back the duodenum, the inferior vena cava and the mesenteric vessels. The intra hepatic biliary tree, the common bile duct(15 mm) and the pancreatic duct (4 mm) were dilated.It was impossible to determine whether it was a pancreatic or duodenal mass (Fig. 2). The pre and post-contrast computed tomography (CT) showed a smooth-outlined solid mass (52 mm × 35 mm) in the second part of the duodenum with a peripheric enhancement. The mass was compressing the pancreatic head. The intrahepatic biliary tree, the common bile duct (17 mm), and the pancreatic duct (5 mm) were dilated. The pancreas was otherwise normal. Neither lymphadenopathy nor metastasis was observed (Fig. 3).

Based on these preoperative findings, the diagnosis was an ampullary of vater tumor. There was no evidence of either extra pancreatic or peritoneal metastasis. The patient was operated Pancreatoduodenectomy was done. Intra-operative exploration found a white nodular tumor with areas of necrosis and measured 5.5 × 4.5 cm. It arises from the ampulla of Vater without involvement of pancreas. The tumor was removed completely with negative margins. Microscopic and immunohistochemical studies revealed spindel-shaped cells that were positive for CD 117 and Vimentin. The mitotic index was 1 mitosis per 50 high-power fields, which classified the patient in the intermediate-risk group, according to the National Institutes of Health (NIH) consensus criteria for risk stratification of GISTs. The final diagnosis was benign STAV and our patient was not treated with imitanib. The post-operative course was uneventful and the patient left the hospital 10 days after operation. One year later, she was totally asymptomatic. At the time of this report, 9 years after her operation, the patient was well without tumor recurrence, although careful follow-up is required with ultrasound and CT. The work has been reported in line with the SCARE 2020 criteria [2].

## 3 Discussion

GISTs represent a wide clinical spectrum of tumors with different locations, which may arise throughout the entire gastrointestinal tract. They are located typically in the submucosa of the stomach and the small and large bowels. Duodenal GIST is even rarer representing only 4 % of these tumors [3]. STAV is an extremely scarce entity [1], as only 17 cases have been described to date in literature according to a Medline search using the key words “ampulla of Vater” and “gastrointestinal stromal tumor”. The analysis of a sample of 18 cases (17 cases collected in the medical literature and our case) allowed us to conclude that STAV is a tumor of adult after the age of fifty. The patients in the published reports were eight men and nine women between 36 and 83 years of age. Our case was the youngest among these (21 years old). The tumor size ranged from 1.6 to 9 cm and the average was 4.3 cm. STAV presents a large clinical spectrum mimicking symptoms of adenoma and adenocarcinoma of the ampulla of Vater. It can be responsible for epigastric pain, jaundice with hyperbilirubinemia, gastrointestinal bleeding with anemia, nausea and vomiting. Radiological investigations usually performed to explore an epigastric pain and jaundice. US has a diagnostic low-yield. In fact, in this review, US was able to show the tumor in 2 cases in which it was impossible to determine whether it was a pancreatic or hepatic mass. However, CT, which had a poor diagnosis role in adenocarcinoma of the ampulla as it showed the tumor in only 25 % of cases, seemed to be helpful: it revealed the tumor in the majority of cases (not used in one case) but it was able to identify the tumor as an ampullary one, in only 3 cases of them. MRI of ampullary GIST has been reported to show low signal intensity on T1-weighted images and high signal intensity on the T2-weighted image. In our case, MRI wasn't performed. STAV is a submucosal ulcerating tumor of the duodenal ampullary region that's why biopsy studies are useful (the tumor cells are exposed at the ulcerating lesions). In 13 cases, the diagnosis of GIST was made by the study of biopsies collected endoscopically from the ulcerating lesions (72 %). Endoscopic ultrasonography (EUS) and Fine needle aspiration (FNA), a technique known as EUS-FNA, is a reliable and robust tool, that enhances the possibilities for early diagnosis of GISTs [3]. EUS demonstrated typically a round, low-echoic mass originating from the muscularis propria. Despite the fact that EUS-FNA is considered to be the first choice in these cases, several limitations regarding the difficulties encountered even by specialists in collecting adequate amount of tissue samples during the process [3] also some centers do not perform EUS so the diagnosis is established mainly postoperatively by histological examination of the excised material [4]. The differential diagnosis may include neuroendocrine tumor, gangliocytic paraganglioma, and intra-ampullary-type carcinoma, and there are also a few case reports of leiomyoma and leiomyosarcoma. The gold standard for treatment of STAV is surgical resection without rupture of the capsule [5]. If technically possible, local resection may be considered [6], [7]. However, when the location of the lesion present challenge, a pancreatoduodenectomy should be performed for STAV [5]. GIST rarely metastasize to lymph nodes and therefore regional lymphadenectomy is generally not required nevertheless, there has been one documented case of STAV presenting with multiple liver and lymph node metastasis [8], [9]. Previous investigators have confirmed that tumor size and mitotic rate correlate strongly with the clinical behavior of GIST [5]. In general, adjuvant therapy with a tyrosine kinase inhibitor is recommended for patients with high-risk tumors, with a tumor size >5 cm, mitotic count >5/50 HPF according to the NIH consensus criteria for risk stratification of GIST [4], [10]. In our case, the patient underwent no adjuvant therapy because she was in the intermediate risk group. However, non-gastric GISTs are associated with less favorable outcomes than the gastric one (modified NIH criteria 2008) [11]. The patient in this case report, has been doing well without recurrence during the 9 years, since surgery, and we will continue to monitor her with a strict follow-up.

## 4 Conclusion

GIST rarely develops in the duodenal ampulla region and it has no specific symptomatology. Radiological investigations play an important role in the diagnosis of STAV mainly CT because STAV has a large size in the majority of cases contrary to adenocarcinoma. However, EUS with biopsies and immunoassaying allows positive preoperative diagnosis in the majority of cases. The gold standard for treatment of STAV is surgical resection without rupture of the capsule.

**Table:** 

Gender	Size	US/MRI	CT	Diagnostic by	Assessment of extension	Surgery	Malignancy	Reference	Year
M	2	(−)	Dominant ampullary mass	Surgery		No indicated	No indicated	[20]	2019
F	2.3	MRI: an enhancing 1.7 × 1.5 cm mass within the periampullary region	Common bile duct dilation and 1 cm soft tissue nodule with peripheral enhancement within the third portion of duodenum	Surgery	(−)	Open pancreatic sleeve duodenectomy	Low risk	[19]	2017
F	3	(−)	An enhancing, 1.9 × 2.1 cm mass was found in the periampullary region protruding into the second portion of the duodenum	EUS	(−)	Local resection	Low risk	[5]	2016
M	2.2	MRI: The mass showed low signal intensity on T1-weighted images and high signal intensity on T2-weighted images	A smooth-outlined hypervascular solid mass in the second part of the duodenum.	EUS	(−)	PD	Low risk	[1]	2014
M	1.6	US/MRI: no dilatation	A 2 cm hypervascular lesion with a regular border lying between the head of the pancreas and the second part of the duodenum. No dilatation	Endoscopic enucleation	(−)	Local resection	Low risk	[7]	2013
F	3.0	(−)	Hypervascular Mass infiltrated the wall of the duodenum without obstruction of the lumen	Endoscopy	(−)	PD	Low risk	[12]	2013
F	3.2	(−)	A solid lesion in the pancreatic head. Neither biliary nor pancreatic duct dilatation was observed and no involvement of the nearby vasculature was noted	Surgery	(−)	PD	Low risk	[4]	2012
M	7.6	(−)	A mass in the duodenal second portion invading the pancreatic head and inferior vena cava	EUS	Invasion of pancreas and the inferior vena cava	PD	High risk	[13]	2010
F	4.5	US: A 5 cm hypoechogenic image that had lobulated limits and contained central calcifications. It was pushing back the duodenum, the inferior vena cava and the mesenteric vessels. It was impossible to determine whether it was a pancreatic or hepatic mass.	A solid, hypodense pancreatic mass with a peripheric enhancement that contained central calcifications	Surgery	(−)	PD	Low risk	[14]	2009
M	2.6	(−)	Duodenal wall thickening in the proximal and mid –descending duodenum	EUS	(−)	Local resection	Low risk	[6]	2007
M	3.0	(−)	Heterogeneous enhancing tumor in the ampullary region	EUS	(−)	PD	–	[8]	2007
M	9.0	(−)	Primary tumor in the second part of the duodenum, compressing the pancreatic head. The intra hepatic biliary tree, the main duct channel (8 mm) and the pancreatic duct were minimally dilated. The pancreas was otherwise normal	Endoscopic retrograde cholangiopancreatography (ERCP)	(−)	PD	High risk	[15]	2007
F	6.0	(−)	A uniformly enhanced smooth outlined solid mass of the 2nd part of the duodenum (in the ampulla of Vater) with a central niche, which partially obstructed the duodenal lumen. The tumor covered the distal end of the common bile duct and was clearly separated from the pancreatic head and the inferior vena cava;	ERCP	(−)	Local resection	High risk	[3]	2006
M	8.0	US: Dilatation of intra and extra hepatic biliary tracts and pancreatic duct, hepatic tumor and portal vein thrombosis	Dilatation of intra and extra hepatic biliary tracts and pancreatic duct, hepatic tumor and portal vein thrombosis	EUS	Pancreatic invasion/liver metastasis/lymph nodes	Dead	High risk	[9]	2005
F	4.5	US/MRI: mass of the head of pancreas and dilatation of the biliary and pancreatic ducts	(−)	ERCP	(−)	PD	High risk	[16]	2004
F	5.5	(−)	Smooth outlined solid mass of the duodenum with a central niche, which partially obstructed the duodenal lumen	ERCP	(−)	PD	High risk	[17]	2004
F	4.0	(−)	A uniformly enhanced tumor that was clearly separated from the pancreatic head and the inferior vena cava.	ERCP	(−)	PD	High risk	[18]	2001

## Source of funding

None.

## Author contribution

Wissal Skhiri: Writing - original draft

Jamal Saad: Investigation

Ines Mazhoud: Resources, collecting the data

Mohamed Maatouk: Patient follow up

Asma Chaouch: Visualization

Amina Ben Salem: Validation, supervision

## Ethical approval

I declare on my honor that the ethical approval has been exempted by my establishment.

## Consent

Written informed consent was obtained from the patient for publication of this case report and accompanying images. A copy of the written consent is available for review by the Editor-inChief of this journal on request.

## Guarantor

Not applicable for our manuscript.

## Research registration number

Not applicable.

## Declaration of competing interest

None of the authors have any conflict of interest.
